# Translation of the working alliance inventory short revised into Italian using a Delphi procedure and a forward-backward translation

**DOI:** 10.3389/fmed.2023.1236273

**Published:** 2024-01-11

**Authors:** Nicola Buono, Béatrice Sassier, Hans Thulesius, Robert Hoffman, Patrice Nabbe, Davorina Petek, Jean Yves Le Reste

**Affiliations:** ^1^National Society of Medical Education in General Practice (SNAMID), Caserta, Italy; ^2^ERA 7479, Departement de Mèdecine Generale, SPURBO Universite de Bretagne Occidentale, Brest, France; ^3^Department of Clinical Sciences in Malmö, Centre for Primary Health Care Research, Lund University, Malmö, Sweden; ^4^Department of Family Medicine, Tel Aviv University, Tel Aviv, Israel; ^5^Department of Family Medicine, Faculty of Medicine, University of Ljubljana, Ljubljana, Slovenia

**Keywords:** therapeutic alliance, forward-backward translation, Delphi procedure, Italian general practice, WAI-SR Italian

## Abstract

**Introduction:**

Enhancing treatment adherence, especially for chronic diseases, can be achieved through therapeutic alliance, potentially elevating the quality of care. An instrument to evaluate the therapeutic alliance could be beneficial in routine clinical settings, educational environments, and extensive research efforts at national and European levels. In this study, we translated therapist and patient versions of the Working Alliance Inventory Short Revised (WAI-SR) into Italian.

**Methodology:**

An email-based Delphi method was employed for the English-to-Italian translation, incorporating a forward-backward process. The initial translation team comprised two Italian family physicians proficient in English, a linguist, and a psychiatrist. The forward translation was then reviewed by 18 Italian family physicians through a Delphi process and was subjected to a backward translation by two Italian English teachers. A cultural correspondence was subsequently identified to adjust translations within a national and international framework.

**Results:**

All 18 experts fully engaged in the Delphi process, and consensus was achieved by the second Delphi round. A cultural check checked for discrepancies regarding linguistic consistency with other translations and found no difference.

**Conclusion:**

This Italian translation of the WAI-SR is expected to support Italian family physicians aiming to enhance their clinical practice and therapeutic outcomes. It could also be a valuable tool for Italian medical students to foster therapeutic relationships and improve their communication skills.

## 1 Introduction

The medical practice model has changed during the last century in European countries and worldwide. From a paternalistic physician-centered model, in which the physician holds the knowledge and decides what is suitable for the patient, the physician/patient relationship has moved to a patient-centered, shared decision-making and relational model in which the patient becomes involved in their medical management (1) encouraged to play the role of a partner and to take greater responsibility for their own health (2).

The relational model emphasizes the importance of relationships, interactions, and connections among individuals, which can lead to improved decision-making, enhanced cooperation and better outcomes between patients and medical professionals. Prusiński (2023) sheds light on the regulatory potential of procedural justice in medical advice compliance and treatment adherence, referring to the fairness and transparency of decision-making. Patients who perceive their doctors as respectful, unbiased, and inclusive in decision-making are more likely to adhere to medical advice. This highlights the role of the relational model in healthcare outcomes (3).

Similarly, Prusiński’s earlier work in 2022 delves into the influence of a doctor’s authority on patients’ treatment decisions. The research underscores that the relational dynamics between doctors and patients play a pivotal role in shaping patients’ perceptions of medical authority. When doctors establish empathetic and collaborative relationships with patients, it can lead to shared decision-making, increased patient satisfaction, and improved adherence to treatment plans. This aligns with the relational model’s emphasis on communication, trust, and shared understanding (4, 5).

In shared decision-making, it is crucial to build a suitable relationship with the patient to facilitate the sharing of information, which will permit patients to deliberate with their therapist and express their preferences and points of view (6). This alliance between physician and patient is termed the “Therapeutic Alliance,” describing how patients and therapists work together to accomplish specific goals (7).

A common definition of therapeutic alliance comes from Bordin (1979) on the generalizability of the concept of working alliance (8), where he outlined the three elements or ‘dimensions’ that make up a therapeutic alliance: the relationship between the patient and the therapist – ‘development of bonds’; their shared understanding of the therapy’s objectives – ‘agreements on goals’; and their shared understanding of the activities involved in the treatment – ‘assignment of tasks’.

Bordin stressed the concept’s psychodynamic origin, but his intention with the definition was to transform it into a pan-theoretical construct. Research spanning several decades consistently highlights the significance of the therapeutic alliance in predicting positive therapy outcomes across various therapeutic approaches and client populations. A robust therapeutic alliance contributes to better engagement in therapy, increased adherence to treatment recommendations, and enhanced client satisfaction (6, 7, 9, 10).

The concept of alliance in therapy emphasizes the importance of a strong working relationship between patient and therapist (5, 8). Although the field has yet to settle on a uniform definition of the concept (9), there seems to be convergence empirically and theoretically that the central aspects of the therapeutic alliance construct involve the bond between the therapist and the patient as well as agreement about the therapeutic goals and tasks (6, 8, 10–13).

Building and maintaining a therapeutic alliance becomes even more crucial in aging populations and individuals with chronic diseases, who often require ongoing management. It’s not just about prescribing medications and treatments; it’s about creating a comprehensive and supportive framework that helps patients lead healthier lives while effectively managing their conditions (14).

On the other hand, non-compliance with medical advice is generally attributed to the patient’s problem (depression, disturbances of cognitive functions, lack of motivation, rejection, cultural issues, alternative systems of beliefs) without a critical analysis of relational factors (4, 15).

The most common paradigm in medical education continues to be disease-oriented, hospital-based, and more concerned with treatment and healing than caring (16). This has led medical students to develop negative attitudes toward chronic diseases throughout their curriculum and training, even where students did not hold these attitudes before attending medical school (17). Thus, Medical schools must modify curricula to care for patients with chronic diseases (18). This could be achieved by developing skills to enhance the physician-patient relationship and improve the therapeutic alliance.

Several methods exist for evaluating the therapeutic alliance (19–21). An Italian version of the Working Alliance Inventory (WAI) has been available since 2002, but the WAI-SR has yet to be translated into Italian (22). The shorter WAI-SR makes it more suitable for a primary care setting, which in the Italian context is characterized by varying degrees of health literacy and a fast-paced nature of the consultations. To ensure valid international comparisons, translated versions of the questionnaire must possess similar characteristics to the original. This includes adhering to the original language regarding concepts (23) and semantics, which means that the concepts being measured by the scales should exist in different cultures, and the wording of the questions should have equivalent meanings in other languages (24). In international research, these scales need to be culturally equivalent, ensuring that people from different cultural backgrounds can understand, interpret, and assess the subject matter in a way that is similar or equal across cultures.

The procedure for assessing cultural linguistic equivalence involves several steps:

- Back-translation: The translated version of the scale is evaluated by translating it back into the original language to check for any discrepancies or loss of meaning.
- Test–Retest: Bilingual respondents are used to assess the reliability of the translated version through test–retest procedures.
- Adaptation: The translated version may need further adaptation to accurately capture the intended concepts and meanings in the target culture.
- Cultural Check: Finally, a principal researcher in the target country conducts a cultural check to ensure that the scale is culturally appropriate and meaningful within the context of that culture. This process helps researchers ensure that assessment scales are translated accurately, culturally relevant, and applicable, allowing for meaningful and valid cross-cultural comparisons in psychological research (25).

A ‘working alliance’ might be interpreted differently across cultures. Italy, with its strong emphasis on interpersonal relationships and familial ties, might interpret or value certain aspects of the therapeutic relationship differently than English-speaking cultures. A direct translation without considering these nuances could miss or misconstrue essential facets of the therapeutic relationship in an Italian setting.

A validated tool in one’s native language can lead to more accurate self-reports, improving patient outcomes. To our knowledge, there was a lack of validated tools in Italian to measure the therapeutic alliance, specifically in primary care. Translating and validating the WAI-SR would fill this gap, providing Italian general practitioners with a tool tailored to their context. Researchers and clinicians can make cross-cultural comparisons with standardized instruments available in multiple languages. Regarding cultural differences, Italians might place a higher emphasis on relational aspects, trust, and rapport. Such nuances were considered during the translation process to ensure that the Italian version of the WAI-SR is both culturally relevant and valid (26).

Our study sought to authenticate and finalize the Italian translation of the English WAI-SR and WAI-SR-T questionnaires in a primary care context. We opted to employ a Delphi process accompanied by a backward translation. This research was part of the “tool assessment for therapeutic alliance study” (TATA study) project, which aims to identify the most validated scale for measuring therapeutic alliance in Europe and translate it into all European languages.

## 2 Materials and methods

### 2.1 Design of the study

The authors participated In establishing an international team of researchers from the European general practice research network (EGPRN). This team, comprised of 10 national research groups, was spearheaded by scholars from The University of Brest, France. This EGPRN team and The University of Brest used a *RAND-UCLA appropriateness method* to find the most appropriate tools to evaluate therapeutic alliance according to its reproducibility and reliability for French and European general practice. The short, revised version of The working Alliance inventory for patient (WAI-SR) and therapist (WAI-SR-T) was selected by 220 GP researchers from 10 European countries between 2013 and 2016 (27–30).

To extend the study and evaluation of the therapeutic alliance within Europe, we needed to validate the WAI-SR and WAI-SR-T translations in other European languages. This paper presents the Italian segment of the study.

We used a Delphi method to ensure semantic, idiomatic, experiential, and conceptual correspondence in the translation. The survey questionnaires and scoring key were translated via email, employing a forward-backward translation process. The translated version was then modified within an Italian context to guarantee the uniformity and cultural correspondence of the questionnaire.

### 2.2 Instrument

The WAI-SR* scale In this study (Paap, 2017) includes a 12-item questionnaire for the patient and a 10-item questionnaire for the physician, evaluating the three key aspects of the physician-patient therapeutic alliance: goal, tasks, and bond (19). Each of the 10 or 12 items is rated on a 5-point Likert scale, ranging from “1: rarely or never” to “5: always.” a high score indicates a more effective therapeutic relationship. A scoring key provides guidance on how to assess the questionnaires.

### 2.3 Participants

**Figure 1:**
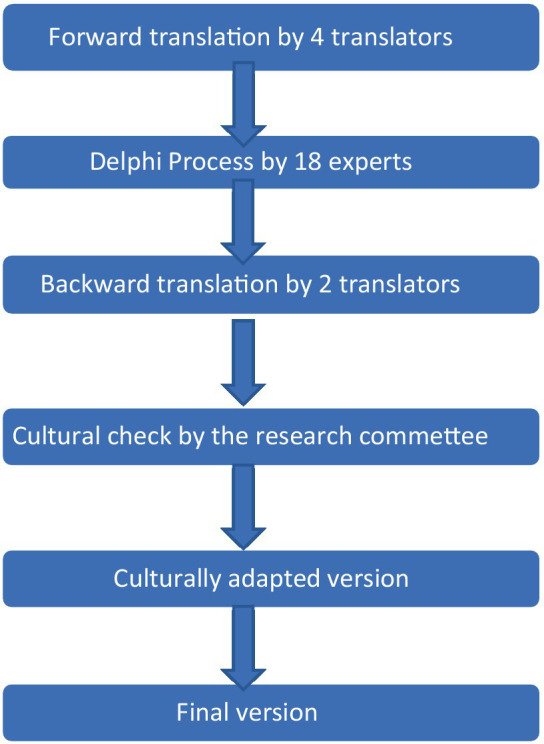
Translation and cross-cultural adaption process flow chart for the WAI-SR into Italian.

To achieve a consistent translation, we adopted a multistep approach to translating the WAI-SR questionnaires systematically (31), as illustrated in Figure 1.

#### 2.3.1 Forward translation by 4 translators

A single investigator assembled a translation group responsible for translating the WAI-SR and the WAI-SR-T from English to Italian. This group comprised individuals proficient in English and native Italian speakers (32). It included two Italian-speaking family physicians with intermediate and upper-intermediate levels of English proficiency, a native Italian-speaking English teacher, and a fluent English psychiatrist. The four experts made a forward translation from English to Italian of both WAI-SR scales. All the translation differences were reconciled until they reached a consensus.

#### 2.3.2 Delphi process

The Delphi method has been used in various fields of research, such as medicine (33), nursing (34), and ecology (35). It Is a qualitative method aimed at reaching a consensus about a subject by independently consulting group members. This way, an attempt is made to counteract the usual group dynamics with highly dominant persons. Members of the Delphi process are called “experts.” this term means that every member has practical, political, legal or administrative knowledge about a specific subject and sufficient legitimacy to express a representative opinion within their area of expertise. The number of experts participating in a Delphi process is not predetermined. The quantity depends on the scope of the problem and the resources available (36). Still, having at least 10 experts at the end of the process is recommended, which means inviting more than 20 at the start to allow a margin for refusals and dropouts.

#### 2.3.3 Recruitment of expert family physicians

The same researcher who recruited the translation group recruited a convenient sample of 20 Italian family physicians natives who voluntarily agreed to participate in the Delphi method procedure as experts to achieve a consensus regarding the translation. The family physicians were contacted anonymously and separately by e-mail. All participants were provided with a written explanation of the aims and procedure of the study and signed a statement on voluntary participation. Among those twenty invited experts, two declined to participate.

#### 2.3.4 Validation of the forward translation by the Delphi method

To establish a semantic correspondence, both questionnaires were emailed to 18 specialist Italian family physicians in alignment with the Delphi method. This included a sequence of judgments in an iterative process and monitored feedback of opinions (37). Each physician expert was asked To approve or reject the translation by rating each sentence on a scale of 1–9, with 1 signifying “no agreement” and 9 representing “full agreement.” if a translation was rated lower than 7, they were prompted To explain their disagreement and suggest a more appropriate translation.

Validation for each statement was considered successful if at least 70% of the participants rated it 7 or higher. If a statement did not meet this standard, the lead researcher proposed a new translation, considering the family physician experts’ recommendations. This revised translation was once more distributed to the group for a second round of the Delphi method. This process was continued until every statement was validated to satisfaction.

We needed two Delphi consensus procedures to reach an agreement among the experts in the present study.

#### 2.3.5 Backward translation

Two Independent translators, Italian instructors of advanced English at a public secondary school in Italy, translated the Italian versions of the WAI-SR, WAI-SR-T, and the scoring key back into English. The goal of the backward translation was to create a linguistic counterpart of the original document that accurately reflected the same meaning. Any inconsistencies in the translation were deliberated with the two back translators, and additional translation efforts were made until an acceptable version was finalized. Consensus was reached after a single round of the agreement procedure between the two expert translators for both WAI SR scales. However, getting a consensus for the back-translation of the scoring key required two rounds.

#### 2.3.6 Cultural check

During a workshop at the EGPRN meeting in Dublin in 2017, the global TATA research group, comprising all national supervisors, international supervisors and two linguists, convened biannually for 2 years, reviewed translation obstacles and compared the back-translated versions with the original English versions. Leaders from five country teams and an international committee led by the principal investigator of the TATA group conducted a cultural assessment by comparing back-translations of five languages, including Italian, with the original versions of the WAI-SR and WAI-SR-T. The primary objective was to identify any translated items where the meaning was lost or inaccurately altered during translation. If an issue could not be resolved, it was delegated to a local research team to formulate a solution.

### 2.4 Information and consent

Every participant in the study received information that explained the aim and the procedure of the research, and each participant completed and signed a consent form.

#### 2.4.1 Legal requirements

Professor Adam O. Horvath of Simon Fraser University (Canada) and Professor Robert L. Hatcher of the University of Michigan (USA) permitted us to use the WAI for this research (December 20, 2015).

## 3 Results

### 3.1 Composition of the translation group

The translation team comprised two Italian family physicians proficient in English, one linguist, and one psychiatrist. The group consisted of three males (the two family physicians and the psychiatrist) and one female (the linguist), with an average age of 55 years (ranging between 43 and 60 years). Both family physicians were practicing in a rural group setting; the psychiatrist was employed at a hospital, and the linguist served as an English teacher at a university. All members were fluent in English. A written agreement was signed by all four experts. Detailed characteristics of the group members can be found in Table 1.

**Table 1:** WAI SR forward Italian translation group.

Profession	Reading	Level of English		Number of scientific publications in English
		Writing	Speaking	
Family physician	Intermediate	Intermediate	Intermediate	14
Family physician	Upper-intermediate	Upper-intermediate	Upper-intermediate	20
Linguist	Advanced	Advanced	Advanced	/
Psychiatrist	Intermediate	Intermediate	Intermediate	1

The ‘intermediate level’ is equivalent to the B1 level of the Common European Framework of Reference for Languages (CEFR), the “Upper-Intermediate” level is equal to the B2 level of the CEFR, and the “Advanced” level is equivalent to the C1 level of the CEFR.

### 3.2 Characteristics of the expert GPs

The local investigator recruited expert family physicians separately by e-mail from September to October 2016. Eighteen experts participated in the entire study after two had been lost to follow-up. Three family physician experts were women (17%), and 15 were men (83%). The average age of participants in the study was 62 years, and the average number of years in practice was 34 years. Most family physician experts worked in group practices (61%) and urban environments (67%), and most had teaching and research responsibilities. 56% of experts had a fluent English level, 11% an intermediate level, and 33% a basic level.

### 3.3 Delphi process

#### 3.3.1 WAI-SR patient scale

For an item to be validated, at least 13 expert participants had to rate it as 7 or higher. The Delphi round for the WAI-SR patient scale yielded acceptable agreement among all expert participants. Consensus was reached, with over 70% of responses scoring 7 or higher for each item on the WAI-S (Table 2).

**Table 2:** WAI-SR patient scale Likert scores, mean and median – Round 1 (*N* = 18).

Results	Inst	Ans	Q1	Q2	Q3	Q4	Q5	Q6	Q7	Q8	Q9	Q10	Q11	Q12
≥7 (n/18)	18	18	16	17	15	17	16	15	17	14	16	17	15	18
≥7 (%)	100	100	88	94	83	94	88	83	94	78	88	94	83	100
Mean	7.7	8.1	8.2	8.0	7.9	7.7	8.2	8.0	8.1	7.6	7.9	7.9	7.9	7.9
Median	8	8	8	8	8	8	8	8	8	8	8	8	8	8

#### 3.3.2 WAI-SR physician scale

The Delphi round for the WAI-SR physician scale demonstrated acceptable agreement in all participants. A consensus was achieved, with more than 70% of responses scoring 7 or above for each WAI -SR item (Table 3).

**Table 3:** WAI-SR therapist scale Likert scores, mean and median – Round 1 (*N* = 18).

Results	Inst	Ans	Q1	Q2	Q3	Q4	Q5	Q6	Q7	Q8	Q9	Q10
≥7 (n/18)	18	18	16	16	17	17	14	15	17	14	16	17
≥7 (%)	100	100	88	88	94	94	78	83	94	78	88	94
Mean	7.8	8.0	7.8	7.9	8.1	7.7	8.1	7.9	7.6	8.1	7.8	7.8
Median	8	8	8	8	8	8	8	8	8	8	8	8

N, number of participants that rated the item ≥ 7 out of all 18 participants; Inst, instructions; Ans, Likert scale answers; Q, question.

#### 3.3.3 WAI-SR scoring key scale

The scoring key provides instructions for the evaluation of the scale. The translation process of the scoring key followed the same approach as that used for the WAI-SR items. It was validated over two Delphi rounds, with question 6 (Q6) receiving a score of 6 from two out of 18 individuals (11%) and all other questions, except Q11, scoring above 7 (Table 4). Q11 was modified following a consultation with the scale’s author, AO Horvath, for additional guidance.

**Table 4:** WAI-SR scoring sheet scale.

Results	Q1	Q2	Q3	Q4	Q5	Q6	Q7	Q8	Q9	Q10	Q11
≥7 (n/18)	16	17	15	17	16	15	17	14	16	17	15
≥7 (%)	88	94	83	94	88	83	94	78	88	94	83
Mean	7.9	8.0	8.4	7.7	8.2	7.6	8.0	8.2	8.4	8.4	8.2
Median	8	8	9	8	8	8	8	8	9	9	8

Likert scores, mean and median. Q1, scoring key; Q2, patient scale mean; Q3, family physician scale mean; Q4, scale type; Q5, goal; Q6, task; Q7, bond; Q8, WAI-SR item; Q9, score patient version; Q10, score family physician version; Q11, Instructions.

### 3.4 Backward translation

The backward translation emphasized conceptual and cultural equivalence and found no discrepancies. If a difference in the forward- backward translations changed the meaning of the item, then the difference was considered ‘significant,’ otherwise ‘insignificant.’

Insignificant differences are shown in black, and significant differences are shown in red in the Supplementary Tables S4–S6.

### 3.5 Comprehensive cultural equivalence evaluation

The cultural equivalence process identified a few potential translation challenges:

In the instructions for the “WAI SR Patient scale,” the original English version utilized the term “therapist.” However, the forward and backward translations employed the word “family physician.” The Italian version preserved this term, translated as “medico di famiglia/terapista.” Given the work conducted by local family physicians in translating, validating, and evaluating the scales, we concluded that their suggested translation was appropriate.

In the instructions for the “WAI SR Physician scale,” the original English version referred to “people and client.” However, the backward translation used “family physician and patient.” Consequently, in the final Italian translation, the term “therapist” was retained for consistency with the original English title of the WAI-SR Physician scale.

In the scoring keys for Q5 and Q6, about translating “goal” and “task,” two family physicians (11%) translated these words into Italian as “scopo” for Q5 and “lavoro” for Q6. However, the linguistics expert argued that there was no cultural equivalence, and as a result, the translations “compito” and “obiettivo” were preserved in the final version (Table 4).

A discrepancy occurred in the translation of the sentence explaining the usage of the scoring sheet. The original English version stated, “To derive a scale or total score, simply sum and take the mean of the items.” The backward English version was “To obtain a scale or a total score, just sum up or take the mean of the numbers.” The Italian translation presented a mismatch in meaning, which was adjusted from “Per ricavare una scala o un punteggio totale, semplicemente fai la somma o la media dei numeri” to “Per ricavare una scala o un punteggio totale, semplicemente fai la somma e la media dei numeri.” For further clarification, we consulted the author of the scale, AO Horvath. Q11 was subsequently corrected and referred back to the expert group for revision. Later, consensus was reached for Q11.

After the second Delphi round, the final version of the Italian translation of the WAI-SR, which included the above modifications, was implemented.

## 4 Discussion

### 4.1 Main results

This study accomplished a validated Italian rendition of the WAI-SR and WAI-SR-T questionnaires, potentially assisting Italian family physicians in enhancing care quality, particularly for chronic disease patients. Furthermore, it could be an unbiased tool for instructing medical students in therapeutic alliance. It required two Delphi technique rounds to achieve a consensus on all questionnaire item translations, and after a few minor adjustments, the back-translation achieved cultural equivalence.

The translation and Delphi processes presented diverse challenges, encompassing linguistic and cultural nuances in content translation, potential misinterpretation of complex terms, and the importance of maintaining consistency in terminology and style throughout the translated questionnaire. The Delphi process involved participants from various cultural backgrounds, required culturally sensitive yet unbiased questions and statements, emphasizing the need for consistent phrasing across rounds to ensure reliable results. Following data collection in the Delphi survey, analysis and synthesis were crucial to derive meaningful insights and consensus. Ethical considerations, including informed consent, confidentiality, and sensitive data handling, were integral to the overall process.

### 4.2 Analysis of forward translation

Upon completion of each forward translation, translators convened to resolve discrepancies between their versions. However, certain items incorporated terms or concepts that could be translated differently, depending on the original English text’s intended meaning (for instance, “client,” “toward mutually agreed-upon goals,” and “derive a scale”). Instead of directly translating the term into Italian, a consensus was reached in each case that conveyed the question’s idea. Rather than a straight conversion into Italian, the multilingual expert panel devised a first “reconciliation” Italian version, which they felt best translated the original English text.

### 4.3 Analysis of backward translation

The initial reconciled Italian version was independently back-translated into English by two distinct bilingual English–Italian speakers. The two back-translations were compared and discussed, and their differences from the original were analyzed to determine if any information was lost during translation. Many minimal adjustments arose from various ways of expressing the same idea. Corrections were made in the Italian translation.

### 4.4 Validation process and international comparison

Although complex and time-consuming, the equivalence method used in translating the two measures assessing therapeutic alliance was effective for semantic validation. The same procedure was utilized to validate the WAI-SR questionnaire in other countries (38). A polish translation demonstrated the approach’s practicality, requiring only one Delphi round to achieve consensus (38). The advantage of this procedure is that it was simultaneously taking place in several European countries with different linguistic bases, which provided the opportunity to discuss the difficulties national and local research groups met while translating the original WAI-SR scales. The Delphi method was used to validate the approved forward translation and proved to be suitable for exploring areas of disagreement, contention, or ambiguity. During this process, translations of WAI-SR scales were thoroughly tested with target demographic or language group representatives to ensure their understanding of the questionnaire was consistent with the original. We believe this method was justified for translation, providing a precise consensus (39).

In our view, every questionnaire translation should undergo a cultural equivalence adaptation to identify and correct deficient expressions in the translation and to distinguish any differences between the original and back-translated items. The work of Streiner et al. (40) influenced the process’s earliest stages. Recent guidelines (41, 42) confirmed the standardized strategy for cultural adaptation of patient-measured outcomes. In this study, we followed the recommendations throughout: initially, by using the Delphi method, recognizing it as the best choice given our linguistic, social, and cultural context, and subsequently, by the oversight of researchers led by the University of Brest, who supervised the questionnaire’s adaptation and the cultural adaptation based on back-translation. This ensured items were translated while maintaining their structure and essence.

### 4.5 Strengths and limitations of the study

Information bias was minimized among experts by individually sending documents via anonymized emails. Anonymity also secured the Delphi procedure’s quality, eliminating issues like dominance, conflicts of interest, and group pressures typically associated with expert panels (43).

Moreover, when translating questionnaire scales like the WAI-SR, it’s preferable to have forward translations done by professionals familiar with the questionnaire’s terminology, experienced in translating scales and possessing native language skills. The content of the WAI-SR spans psychology and medicine, requiring its translation to be comprehensible to both physicians and patients. This challenge was mitigated by forming a group of two family physicians, a psychologist, and a linguist to perform the forward translation. The selection of the recruited Italian family physician experts was unbiased, with their English proficiency levels ranging from intermediate to advanced.

We only covered the first stage of a complete validation of the WAI-SR Italian, which involved confirming the semantic and cultural equivalency of the scale. Results of evaluations of item reliability and validity will be presented in further research. To compare response patterns with the scale constructs that have been proposed, exploratory principal component analysis will be performed. Future research should focus on four key areas: the psychometric features of the Italian WAI-SR scale, the scale’s suitability for family physicians, the administrative logistics, and the theoretical underpinnings of scale interpretation in family medicine.

The Delphi group wasn’t representative of the broader Italian family physician community. However, as the Delphi method is qualitative, population representativeness is not necessary.

Lastly, the back-translation would ideally have been performed by an independent translator proficient in Italian but whose first language was English. Due to the lack of such translators, we settled for two independent licensed Italian translators unfamiliar with the WAI-SR scale.

### 4.6 Implication for clinical practice, medical education, and future research

The Italian translation of the WAI-SR could benefit clinical practice, medical training, and further research on therapeutic alliance. In 2016, Italy had the highest proportion (22%) of the population over 65 years in Europe (44). With an aging population, the prevalence of chronic diseases will inevitably rise. The ICON (improving cardiovascular risk profile in older Neapolitans) study included 503 patients >65 years with cardiovascular risk factors and low socioeconomic status (45) and aimed to enhance patient motivation through a positive patient-physician relationship intervention, focusing on lifestyle changes, particularly smoking cessation. The study suggested that improved physician-patient contact positively altered the cardiovascular risk profile in elderly Neapolitans. The working alliance can be quantified In medical care and appears strongly linked with patients’ treatment adherence and satisfaction (46).

Another example where the WAI SR could be useful is in obesity care. In 2015, Italy had a high prevalence of overweight and obese children (47). The WAI-SR could also be used in diabetes care. A survey among patients with type 1 diabetes found that therapeutic alliance assessment with two tools (Helping Alliance Questionnaire–R and WAI-SR) predicted better glycemic control at a one-year follow-up. The HbA1C level at follow-up was negatively correlated with therapeutic alliance (48, 49).

The 21st-century patient-physician relationship should be holistic and patient-centered. This involves effective communication and interpersonal skills such as empathy, understanding and relational versatility, which could be taught and learned (50). A study of Swedish medical students learning communication skills showed that students often felt intrusive when exploring the patient’s psychosocial situation (51). Medical students need to know how to recognize the patient’s feelings, define them, legitimize them, and respect their efforts to deal with emotions. While teaching and learning communication skills occurs, a therapeutic alliance assessment tool such as the WAI-SR can give concrete feedback so medical students can extend their empathy and improve the patient-physician relationship.

Studies from the USA (52) and Greece (53) that investigated patient-centered attitudes in medical students showed that medical students in later years had more physician-centered attitudes than students in their early years. This paternalistic attitude shift during medical training emphasizes the need for redesigning communication skills curricula, perhaps aided by therapeutic alliance assessments.

WAI-SR could help medical students be more patient-centered by assessing their communication skills development using the WAI-SR in different years of their curricula. WAI-SR could also be used in continuing medical education by supplying discussion topics to GPs and other physicians regarding physician–patient relationships.

A validated therapeutic alliance assessment tool in all European languages could enhance further research. Now that we have an Italian version, Italy can be comparable with other European countries in the quality of therapeutic alliance in clinical practice.

### 4.7 Future research

This translated version could be tested on Italian family physicians to assess its usefulness in improving their clinical practice and therapeutic results. It can also be tested on Italian medical students to enhance their communication skills. Further studies will be needed to compare the backward Italian translation with the Slovenian, Polish, Swedish, Hebrew, French, Bulgarian and Spanish backward translations. Eventually, these studies may help identify cultural discrepancies between European countries and jurisdictions and make it possible to adapt translations within their national contexts to ensure homogeneity.

## 5 Conclusion

Therapeutic Alliance plays a significant role in everyday medicine. It enhances the quality of care. Future health professionals need to develop competence in using it. Therapeutic Alliance will also be the focus of studies and research in the coming years.

This study was part of a more comprehensive study named “Tool Assessment for Therapeutic Alliance study” (TATA study), whose aim was to find the most validated scale to measure therapeutic alliance in the whole of Europe and to translate it into all European languages.

The present translation of the WAI-SR in Italian could facilitate Italian family physicians seeking to improve their clinical practice and therapeutic results. It could also be used with Italian medical students to teach therapeutic alliance and enhance their communication skills.

## Data availability statement

The original contributions presented in the study are included in the article/Supplementary material, further inquiries can be directed to the corresponding author.

## Ethics statement

Ethical review and approval was not required for the study on human participants in accordance with the local legislation and institutional requirements. Every participant in the study received information that explained the aim and the procedure of the research, and each participant completed and signed a consent form.

## Author contributions

JR, HT, and DP conceived and designed the experiments. NB and BS analyzed the data. NB, HT, and JR wrote the manuscript. NB and BS performed the study. HT, PN, and DP supervised the performance of the survey. JR, HT, DP, RH, PN, and BS reviewed the manuscript. All authors have read and approved the manuscript.
