# Supplementary material for: Translation of the working alliance inventory short revised into Italian using a Delphi procedure and a forward-backward translation
Source: Front Med (Lausanne). 2024 Jan 11;10:1236273. doi: 10.3389/fmed.2023.1236273 (PMC10808585; doi:10.3389/fmed.2023.1236273)
Supplement: Supplementary file 1 [file Table_1.DOCX]

**Supplementary Material.**

**Table 5.** WAI- SR PATIENT.

| **English Original Version** | **First Italian Version** | **Backward Translation** | **Final Italian Version** |
| --- | --- | --- | --- |
| Instructions: Below is a list of statements and questions **about** experiences people might have **with their** therapy or **therapist.** Some items refer directly to your therapist with an underlined space -- as you read the sentences, mentally insert the name of your therapist in place of ______ in the text. Think about your experience in therapy, and decide which category best describes your **own** experience. | Istruzioni: Di seguito è riportato un elenco di istruzioni e domande sulle esperienze che le persone possono avere riguardo la loro terapia o il proprio Medico di Famiglia. Alcune voci, con uno spazio sottolineato, si riferiscono direttamente al tuo Medico di Famiglia. Mentre leggile frasi, inserisci mentalmente il nome del tuo Medico di Famiglia al posto di ____ nel testo. Pensa alla tua esperienza riguardo la terapia, e decidi quale categoria meglio descrive la tua esperienza. | Instructions: Below is a list of statements and questions **on the** experiences **that** people might have **about their** **therapy** or **their Family Physicians**. Some items, with an underlined space, directly refer to your Family Physician. As you read the sentences, mentally enter the name of your Family Physician in place of ____ in the text. Think of your experience about therapy, and decide which category best describes your experience. | Istruzioni: Di seguito è riportato un elenco di istruzioni e domande sulle esperienze che le persone possono avere riguardo la loro terapia o il proprio Medico di Famiglia/terapista. Alcune voci, con uno spazio sottolineato, si riferiscono direttamente al tuo Medico di Famiglia. Mentre leggile frasi, inserisci mentalmente il nome del tuo Medico di Famiglia al posto di ____ nel testo. Pensa alla tua esperienza riguardo la terapia, e decidi quale categoria meglio descrive la tua esperienza. |

| English Original Version | First Italian Version | Backward Translation | Final Italian Version |
| --- | --- | --- | --- |
| IMPORTANT!!! **Please** take your time to consider each question carefully. | IMPORTANTE!!! Prendete tutto il tempo necessario per valutare attentamente ogni domanda. | Important!!!  Take your time to evaluate each question carefully. | IMPORTANTE!!! Prendete tutto il tempo necessario per considerare attentamente ogni domanda. |

| English Original Version | First Italian Version | Backward Translation | Final Italian Version |
| --- | --- | --- | --- |
| **Seldom**  Sometimes  **Fairly** often  Very often  Always | Raramente  A volte  Abbastanza spesso  Molto spesso  Sempre | **Rarely**  Sometimes  **Quite** often  Very often  Always? | Raramente  A volte  Abbastanza spesso  Molto spesso  Sempre |

| English Original Version | First Italian Version | Backward Translation | Final Italian Version |
| --- | --- | --- | --- |
| 1. As a result of these sessions **I am clearer as to how I might be able** to change. | 1. Come risultato di queste sessioni mi è più chiaro il modo con cui potrei cambiare. | 1. As a result of these sessions **it is clearer to me how I could change.** | 1. Come risultato di queste sessioni mi è più chiaro il modo con cui potrei cambiare. |

| English Original Version | First Italian Version | Backward Translation | Final Italian Version |
| --- | --- | --- | --- |
| 2. What I am doing in therapy gives me new ways of looking at my problem. | 2. Quello che sto facendo nella terapia mi dà un nuovo modo di guardare al mio problema. | 2. What I am doing in therapy gives me **a** new way of looking at my problem. | 2. Quello che sto facendo nella terapia mi dà un nuovo modo di guardare al mio problema. |

| English Original Version | First Italian Version | Backward Translation | Final Italian Version |
| --- | --- | --- | --- |
| 3. I believe _____ likes me. | 3. Credo di_ piacere a____ | 3. I think___ likes me | 3. Credo di piacere a ____ |

| English Original Version | First Italian Version | Backward Translation | Final Italian Version |
| --- | --- | --- | --- |
| 4. _____ and I collaborate **on setting goals** for **my** therapy. | 4. ___ ed io collaboriamo nel fissare gli obiettivi della terapia. | 4. ____and I collaborate **to set the goals** of my therapy. | 4. ___ ed io collaboriamo nel fissare gli obiettivi della terapia. |

| English Original Version | First Italian Version | Backward Translation | Final Italian Version |
| --- | --- | --- | --- |
| 5._____ and I respect each other. | 5. ___ ed io ci rispettiamo. | 5.____and I respect each other | 5. ___ ed io ci rispettiamo. |

| English Original Version | First Italian Version | Backward Translation | Final Italian Version |
| --- | --- | --- | --- |
| 6. _____and **I are working towards mutually agreed-upon goals.** | 6.___ ed io lavoriamo di comune accordo sugli obiettivi | 6. ____and **I work together on the goals** | 6.___ ed io lavoriamo di comune accordo sugli obiettivi concordati. |

| English Original Version | First Italian Version | Backward Translation | Final Italian Version |
| --- | --- | --- | --- |
| 7. I feel **that** _____ appreciates me. | 7. Io credo che___mi apprezza. | 7. I feel ____appreciates me | 7. Io credo che___mi apprezza. |

| English Original Version | First Italian Version | Backward Translation | Final Italian Version |
| --- | --- | --- | --- |
| 8._____ and I agree on what is important **for** me to work on. | 8.____ed io siamo d’accordo su quali sono per me le cose importanti su cui lavorare. | 8.___and I agree on what is important **to** me to work on | 8.____ed io siamo d’accordo su quali sono per me le cose importanti su cui lavorare. |

| English Original Version | First Italian Version | Backward Translation | Final Italian Version |
| --- | --- | --- | --- |
| 9. I feel _____ **cares about me even when I do things that he/she does not approve of.** | 9. Io sento che____si prende cura di me anche quando faccio cose che lui/lei non approva | 9. I feel **confident that_____ takes care of me even when I do things he/she doesn’t approve** | 9. Io sento che____si prende cura di me anche quando faccio cose che lui/lei non approva |

| English Original Version | First Italian Version | Backward Translation | Final Italian Version |
| --- | --- | --- | --- |
| 10. I feel that **the things I do in therapy will help me to accomplish the changes that I want.** | 10. Ho la sensazione che quello che faccio nella terapia mi aiuta a realizzare i cambiamenti che voglio. | 10. I feel **that what I am doing in therapy will help me to make the changes I want** | 10. Ho la sensazione che quello che faccio nella terapia mi aiuta a realizzare i cambiamenti che voglio. |

| English Original Version | First Italian Version | Backward Translation | Final Italian Version |
| --- | --- | --- | --- |
| 11._____ and I have established a good understanding of the kind of changes that would be good **for** me. | 11._____ ed io abbiamo stabilito una buona comprensione del tipo di cambiamento che sarebbe buono per me. | 11. ____and I have established a good understanding of the kind of change that would be good **to** me. | 11._____ ed io abbiamo stabilito una buona comprensione del tipo di cambiamento che sarebbe buono per me. |

| English Original Version | First Italian Version | Backward Translation | Final Italian Version |
| --- | --- | --- | --- |
| 12. I believe the way we are working with my problem is correct. | 12. Io credo che il modo con cui stiamo lavorando sul mio problema sia corretto. | 12. I believe that the way we are working with my problem is correct. | 12. Io credo che il modo con cui stiamo lavorando sul mio problema sia corretto. |

**Table 6.** WAI -SR THERAPIST.

| **English Original Version** | **First Italian Version** | **Backward Translation** | **Final Italian Version** |
| --- | --- | --- | --- |
| Instructions: below is a list of statements about experiences **people** might have with their **client.** Some items refer directly to **your client** with an underlined space as you read the sentences,  mentally **insert the name of your client** in place of _____ in the text. | Instruzioni: Di seguito è riportato un elenco di istruzioni riguardo le esperienze che i terapisti possono avere con i loro pazienti. Alcune voci, con uno spazio sottolineato, si riferiscono direttamente al paziente. Mentre leggi le frasi, inserisci mentalmente il nome del paziente al posto di ___ nel testo. | Instructions: Below is a list of statements about the experiences that **Family Physicians** might have with **their patients.** Some items, with an underlined space, directly refer to **the patient.** As you read the sentences, mentally **enter the patient's name** instead of ___ in the text. | Instruzioni: Di seguito è riportato un elenco di istruzioni riguardo le esperienze che i terapisti possono avere con i loro pazienti. Alcune voci, con uno spazio sottolineato, si riferiscono direttamente al paziente. Mentre leggi le frasi, inserisci mentalmente il nome del paziente al posto di ___ nel testo. |

| English Original Version | First Italian Version | Backward Translation | Final Italian Version |
| --- | --- | --- | --- |
| Important!!! **Please** take your time to **consider** each question carefully. | IMPORTANTE!!! Prendete tutto il tempo necessario per valutare attentamente ogni domanda. | Important!! Take your time to **evaluate** each question carefully. | IMPORTANTE!!! Prendete tutto il tempo necessario per considerare attentamente ogni domanda. |

| English Original Version | First Italian Version | Backward Translation | Final Italian Version |
| --- | --- | --- | --- |
| **Seldom**  Sometimes  **Fairly** often  Very often  Always | Raramente  A volte  Abbastanza spesso  Molto spesso  Sempre | **Rarely**  Sometimes  **Quite** often  Very often  Always | Raramente  A volte  Abbastanza spesso  Molto spesso  Sempre |

| English Original Version | First Italian Version | Backward Translation | Final Italian Version |
| --- | --- | --- | --- |
| 1._____ and I agree **about the steps to be taken** to improve his/her situation. | 1.___ed io siamo d’accordo circa i passi da fare per migliorare la sua situazione. | 1. ___and I agree **about the steps we should take to** improve his /her situation. | 1.___ed io siamo d’accordo circa i passi da fare per migliorare la sua situazione. |

| English Original Version | First Italian Version | Backward Translation | Final Italian Version |
| --- | --- | --- | --- |
| 2. I am **genuinely concerned** for _____’s welfare. | 2. Io sono naturalmente interessato al benessere di_____. | 2. I am **naturally interested** in the welfare of ____________. | 2. Io sono naturalmente interessato al benessere di_____. |

| English Original Version | First Italian Version | Backward Translation | Final Italian Version |
| --- | --- | --- | --- |
| 3. We **are working** **towards mutually agreed-upon goals.** | 3. Noi lavoriamo di comune accordo sugli obiettivi. | 3. We **work together on the goals.** | 3. Noi lavoriamo di comune accordo sugli obiettivi concordati. |

| English Original Version | First Italian Version | Backward Translation | Final Italian Version |
| --- | --- | --- | --- |
| 4._____ and **I both feel confident about the usefulness of our current activity in therapy.** | 4. ___ed io siamo sicuri circa l’utilità del nostro impegno nei confronti della terapia. | 4.____and **I are confident about the usefulness of our commitment to the therapy** | 4. ___ed io siamo sicuri circa l’utilità dell'attività attualmente svolta in ambito terapeutico |

| English Original Version | First Italian Version | Backward Translation | Final Italian Version |
| --- | --- | --- | --- |
| 5. I appreciate _____ as a person. | 5. Io apprezzo____come persona | 5. I appreciate ____________ as a person | 5. Io apprezzo____come persona |

| English Original Version | First Italian Version | Backward Translation | Final Italian Version |
| --- | --- | --- | --- |
| 6. We have established a good understanding of the kind of changes **that  would be good for _____.** | 6. Noi abbiamo stabilito una discreta intesa circa il tipo di cambiamento che andrebbe bene per_____. | 6. We have established a good understanding of the kind of **change that would be fine for____** | 6. Noi abbiamo stabilito una discreta intesa circa il tipo di cambiamento che andrebbe bene per_____. |

| English Original Version | First Italian Version | Backward Translation | Final Italian Version |
| --- | --- | --- | --- |
| 7. _____ and I respect each other. | 7. ____ed io ci rispettiamo. | 7. ___and I respect each other. | 7. ____ed io ci rispettiamo. |

| English Original Version | First Italian Version | Backward Translation | Final Italian Version |
| --- | --- | --- | --- |
| 8._____ and I have a common **perception** of his/her goals. | .____ed io abbiamo una comune percezione dei suoi obiettivi. | 8.___and I have a common **feeling of** his/ her goals. | 8.____ed io abbiamo una comune percezione dei suoi obiettivi. |

| English Original Version | First Italian Version | Backward Translation | Final Italian Version |
| --- | --- | --- | --- |
| 9. I respect _____ even when he/she does things that I do **not approve of.** | 9. Io rispetto_anche quando lui/lei fa cose che non approvo. | 9. I respect him/her even when he/she does things I **do not approve.** | 9.Io rispetto___anche quando lui/lei fa cose che non approvo. |

| English Original Version | First Italian Version | Backward Translation | Final Italian Version |
| --- | --- | --- | --- |
| 10. We agree on what is important for _____ to work on. | 10. Noi siamo d’accordo su quali sono per____ le cose importanti su cui lavorare. | 10. We agree on what is important for_____ to work on | 10. Noi siamo d’accordo su quali sono per____ le cose importanti su cui lavorare. |

**Table 7.** WAI -SR-SCORING SHEET.

| English Original Version | First Italian Version | Backward Translation | Final Italian Version |
| --- | --- | --- | --- |
| Scoring key | Modalità di punteggio | Scoring key | Modalità di punteggio |

| English Original Version | First Italian Version | Backward Translation | Final Italian Version |
| --- | --- | --- | --- |
| Patient scale mean | Media scala paziente | Patient scale mean | Media scala paziente |

| English Original Version | First Italian Version | Backward Translation | Final Italian Version |
| --- | --- | --- | --- |
| Family Physician  scale mean | Media scala medico di famiglia | Family Physician  scale mean | Media scala medico di famiglia |

| English Original Version | First Italian Version | Backward Translation | Final Italian Version |
| --- | --- | --- | --- |
| Scale type | Tipo di scala | Type **of** scale | Tipo di scala |

| English Original Version | First Italian Version | Backward Translation | Final Italian Version |
| --- | --- | --- | --- |
| Task | Compito | Task | Compito |

| English Original Version | First Italian Version | Backward Translation | Final Italian Version |
| --- | --- | --- | --- |
| Goal | Obiettivo | Goal | Obiettivo |

| English Original Version | First Italian Version | Backward Translation | Final Italian Version |
| --- | --- | --- | --- |
| Bond | Legame | Bond | Legame |

| English Original Version | First Italian Version | Backward Translation | Final Italian Version |
| --- | --- | --- | --- |
| WAI SR Item | WAI SR singola voce | WAI SR Item | WAI SR singola voce |

| English Original Version | First Italian Version | Backward Translation | Final Italian Version |
| --- | --- | --- | --- |
| Score Patient version | Punteggio versione paziente | Patient version score | Punteggio versione paziente |

| English Original Version | First Italian Version | Backward Translation | Final Italian Version |
| --- | --- | --- | --- |
| Score Family Physician version | Punteggio versione medico di famiglia | Family Physician version score | Punteggio versione medico di famiglia |

| English Original Version | First Italian Version | Backward Translation | Final Italian Version |
| --- | --- | --- | --- |
| **To derive** a scale or total score, simply sum **and** take the mean of the items. | Per ricavare una scala o un punteggio totale, semplicemente fai la somma **o** la media delle voci. | **To obtain** a scale or a total score just sum up **or** take the mean of the items. | Per ricavare una scala o un punteggio totale, semplicemente fai la somma **e** la media delle voci. |
