# Supplementary material for: Translation of the working alliance inventory short revised into Italian using a Delphi procedure and a forward-backward translation
Source: Front Med (Lausanne). 2024 Jan 11;10:1236273. doi: 10.3389/fmed.2023.1236273 (PMC10808585; doi:10.3389/fmed.2023.1236273)
Supplement: Supplementary file 2 [file Data_Sheet_1.pdf]

Annex A: English version of the consent form.

TATA STUDY

INFORMATION NOTICE, INFORMED CONSENT AND SAMPLING DATA FOR PARTICIPATING EXPERTS

INSTRUCTIONS FOR DELPHI ROUNDS' PARTICIPANTS

WAI SR PATIENT
ITALIAN FORWARD TRANSLATION TABLES
EVALUATION INCLUDED

WAI SR THERAPIST
ITALIAN FORWARD TRANSLATION TABLES
EVALUATION INCLUDED

WAI SR SCORING SHEET
ITALIAN FORWARD TRANSLATION TABLES
EVALUATION INCLUDED

INFORMATION NOTICE informed consent and data for sampling (to translate in your language and input with your logo)

Département Universitaire de Médecine Générale

22, avenue Camille Desmoulins CS 93837 – 29238 – Brest CEDEX 3
Tél : 02 98 01 65 52 – fax : 02 98 01 64 74

UFR Médecine & Sciences de la Santé UBO

INFORMATION NOTICE

International Investigator Senior Coordinator
Name: Le Reste Jean Yves
Address: Département de médecine générale, Faculté de Médecine de Brest, 22, avenue Camille Desmoulins, 29238 Brest cedex 3
International Developer
Département Universitaire de Médecine Générale
22 avenue Camille Desmoulins - 29238 Brest Cedex 3
National investigator senior coordinator
Name: …………………… Address: ……………………
National developer ……………………

Dear Madam or Sir

You are invited to participate in a survey by …………………. (trainee in general practice, GP…). The department of general practice from …………………. is the national developer of that survey. He is responsible for it and assume its organization.

Mrs/Mr …………………. will explain his/her work to you. If you decide to participate you will be asked to sign a consent form. This signature will confirm that you did agree to participate.

1- **Course of study**

A forward backward translation study

2- **Potential risk of study**

There are no risks associated with your participation in this study.

3- **Potential benefits of the study**

There is no potential benefit to this study

4- **Voluntary participation**

Your participation to this study is entirely voluntary.
You are free to refuse to participate and to terminate your participation in the study at any time and without incurring any liability or any injury of this fact and without causing consequences.
In this case you must inform the investigator of your decision
In the event that you withdraw your consent, we will conduct a computer processing of your personal data unless written objection on your part.
During the study, your investigator will notify you, if new facts might affect your willingness to participate in the study.

5- **Obtaining complementary informations**

If desired, Professor Le Reste who can be reached at telephone number: 00 33 298 016 552 at any time can answer all your questions about the study.
At the end of the study, and at your request, your investigator will inform you of the overall results of this research.

6- **Confidentiality and use of medical or personal data**

As part of biomedical research in which the DUMG Brest, Professor Le Reste and your national investigator offer to participate, a treatment of your personal data will be used to analyse the results of research in light of the objective of that study which was presented to you.

To this end, the data collected, including any survey and the data on your lifestyle will be forwarded to the promoter of the research where the data will be processed in this study.

Those data will be anonymized and their identification will be held with a code number.

Staff involved in the study is subject to professional secrecy.

These data may also, under conditions ensuring their confidentiality be transmitted to the national or European health authorities.

Under the provisions of Law you have the right to access and modify. You also have the right to object to the transmission of data covered by professional secrecy.

If you agree to participate in this study, thank you to complete and sign the consent form. You will keep a copy of it.

Département Universitaire
de Médecine Générale

22, avenue Camille Desmoulins CS 93837 – 29238 – Brest CEDEX 3
Tél : 02 98 01 65 52 – fax : 02 98 01 64 74

Consent Form
And Data for sampling

Promoter: Département Universitaire de Médecine Générale – 22 avenue Camille Desmoulins - 29238 Brest Cedex 3

Dr: …………………..
Address: …………………..

Local investigator name

Address: …………………..
University: …………………..

Asked me to participate in a forward backward translation study

I had time to reflect on my involvement in this study. I am aware that my participation is completely voluntary and that the study will entail no additional cost to my charge.

I can, at any time, decide to leave the study without giving reasons for my decision and that it does without consequences.

I understood that the data collected during the research would be protected in accordance to confidentiality. They can only be accessed by persons subject to professional secrecy belonging to the team-investigating physician, mandated by the promoter.

I accept the computerized processing of personal data in accordance with the data protection act. I have been informed of my right to access and rectify data concerning me.

My consent does not absolve the responsibilities of the organizers of this research. I retain all my rights guaranteed by Law.

Data for sampling:

Birth date: dd/mm/yyyy Gender: male/female Number of year in practice: …………..………..years

Practice type: solo group others (precisions)

Setting type : rural (less than 2000 inhabitants) semi-rural (between 2000 and 5000) urban (more than 5000 inhabitants)

Teaching activities yes/no Research activities yes/no

Number of publications in English: …………..………. Number of publications in other languages: …………..……….

English level : basic intermediate fluent (all publishers in English are fluent)

Done in two originals at … …………..…… …., the dd/mm/yyyy.

Name, first name of investigator: …………..………. Name, first name of the interviewee: …………..……….

Signature:

Annex B: Italian Version of the consent form.

Società Nazionale di Aggiornamento per il MMG

Sezione di Caserta, Via E. Moneta 3, Caserta 81100, Italy

INFORMATIVA

International Investigator Senior Coordinator
Name: Le Reste Jean Yves
Address: Département de médecine générale, Faculté de Médecine de Brest, 22, avenue Camille Desmoulins, 29238 Brest cedex 3
International Developer
Département Universitaire de Médecine Générale – 22 avenue Camille Desmoulins - 29238 Brest Cedex 3

National investigator senior coordinator:
Name: Buono Nicola
Address: Via Tartari,5 81010 Prata Sannita (Caserta)

National developer: Buono Nicola

Caro Collega,

Sei invitato a partecipare ad un'indagine organizzata dalla Medicina di Famiglia. Il dipartimento di Medicina di Famiglia (SNAMID) di Caserta è lo sviluppatore nazionale ed è responsabile di questa indagine e ne assume l'organizzazione.

Il collega Nicola Buono ti spiegherà il lavoro da fare. Se decidi di partecipare ti verrà chiesto di firmare il consenso informato. La firma conferma che tu sei d'accordo a partecipare.

1- **Svolgimento dello studio**

Studio di forward-backwards translation.

2- **Potenziali rischi dello studio**

Non ci sono rischi associati alla partecipazione allo studio.

3- **Potenziali benefici dello studio**

Non ci sono potenziali benefici dello studio.

4- **Partecipazione volontaria**

La tua partecipazione è interamente volontaria.

Sei libero di rifiutare di partecipare e terminare la tua partecipazione in qualsiasi momento senza incorrere in nessuna responsabilità o danno per questo fatto e senza causare delle conseguenze.
In questa circostanza informa l'investigatore della tua decisione.
Nel caso in cui tu ritiri il tuo consenso, condurremo un trattamento informatico dei tuoi dati a meno che non ci sia una obiezione scritta da parte tua.
Durante lo studio in tuo intervistatore ti informerà se ci sono nuovi eventi che potrebbero inficiare la tua disponibilità a partecipare allo studio.

5- **Ulteriori complementari informazioni.**

Se desiderato il Prof. Le Reste, che può essere raggiunto al numero di telefono: 00 33 298 016 552 in qualsiasi momento, potrà rispondere alle tue domande riguardanti lo studio.
Alla fine dello studio e a tua richiesta, il tuo intervistatore ti informerà su tutti I risultati della ricerca.

6- **Riservatezza e uso di dati medici e personali.**

Come parte di una ricerca biomedica in cui il DUMG Brest, Professor Le Reste ed il vostro intervistatore nazionale ti offrono di partecipare, il trattamento dei tuoi dati personali sarà usato per analizzare I risultati della ricerca alla luce degli obiettivi dello studio che ti sono stati presentati.
Alla fine I dati collezionati, inclusa qualsiasi indagine e i dati riguardanti il tuo stile di vita verranno inviati al promotore della ricerca dove saranno processati per lo studio.
I datti saranno anonimizzati e la loro identificazione sarà fatta usando una codifica numerica.
Lo staff coinvolto nello studio è soggetto a segreto professionale.
Questi dati, sotto condizione che assicura la loro confidenzialità, potranno anche essere trasmessi alle autorità sanitarie Nazionali ed Europee.
Per norma di legge tu hai la possibilità di accesso e modifica. Hai anche la possibilità di opporti alla trasmissione dei dati coperti da secreto professionale.

Se sei d'accordo a partecipare a questo studio per favore completa e firma il consenso informato.
Potrai tenere una copia di esso.

Département Universitaire
de Médecine Générale

22, avenue Camille Desmoulins CS 93837 – 29238 – Brest CEDEX 3
Tél : 02 98 01 65 52 – fax : 02 98 01 64 74

Consenso informato e raccolta dati

Promotore: Département Universitaire de Médecine Générale – 22 avenue Camille Desmoulins - 29238 Brest Cedex 3

Dr:…………………………………………………………………………………………………
Indirizzo:
……………………………………………………………………………………………………….

Nome dell'intervistatore locale
Indirizzo: ……………………………………..
University:

Mi ha chiesto di partecipare in uno studio di forward backward traduzione.

Ho avuto il tempo di riflettere sul mio coinvolgimento nello studio. Sono consapevole che la mia partecipazione è completamente volontaria e che lo studio non comporterà costi addizionali da addebitare.

Posso decidere in qualsiasi momento di abbandonare lo studio senza spiegare la mia decisione e questo non presenterà nessuna conseguenza.

Ho capito che i dati raccolti durante la ricerca saranno protetti in accordo con la riservatezza degli stessi. Essi potranno essere soltanto analizzati da persone soggette a secreto professionale appartenenti al medici del team di investigazione comandati dal promoter.

Accetto il trattamento computerizzato dei miei dati personali in accordo con le misure di protezione dei dati. Sono stato informato della possibilità di accedere e rettificare i miei dati personali.

Il mio consenso non assolve le responsabilità degli organizzatori della ricerca. Io assumo che tutti i miei diritti siano garantiti dalla legge.

Data della raccolta dati:

Data di nascita:

Sesso:

Numero di anni di pratica:

Tipo di organizzazione dello studio di medicina (solo, in gruppo, altro)

Setting dello studio di medicina (rurale (meno di 2000 abitanti), semi rurale (tra 2000 e 5000, urbano (più di 5000 abitanti):

Attività di Insegnamento (Si,No)

Attività di ricerca (Si,No)

Numero di pubblicazioni in Inglese:

Numero di pubblicazioni in altra lingua:

Livello di Inglese: basale, intermedio, fluente (tutti quelli che pubblicano in Inglese hanno in livello fluente…)

Stampato in due copie originali

In…………….. il………………..

Nome e cognome dell'intervistatore: Nome e cognome dell'intervistato:

Firma:

Annex C. WAI SR Patient (English version)

Working Alliance Inventory-Short Revised (WAI-SR)

Instructions: Below is a list of statements and questions about experiences people might have with their therapy or therapist. Some items refer directly to your therapist with an underlined space -- as you read the sentences, mentally insert the name of your therapist in place of ______ in the text. Think about your experience in therapy, and decide which category best describes your own experience.

IMPORTANT!!! Please take your time to consider each question carefully.

1. As a result of these sessions, I am clearer as to how I might be able to change.

□	□	□	□	□
Seldom	Sometimes	Fairly Often	Very Often	Always

2. What I am doing in therapy gives me new ways of looking at my problem.

□	□	□	□	□
Always	Very Often	Fairly Often	Sometimes	Seldom

3. I believe___likes me.

□	□	□	□	□
Seldom	Sometimes	Fairly Often	Very Often	Always

4. ___and I collaborate on setting goals for my therapy.

□	□	□	□	□
Seldom	Sometimes	Fairly Often	Very Often	Always

5. ___and I respect each other.

□	□	□	□	□
Always	Very Often	Fairly Often	Sometimes	Seldom

6. ___and I are working towards mutually agreed-upon goals.

☐ Always ☐ Very Often ☐ Fairly Often ☐ Sometimes ☐ Seldom

7. I feel that___appreciates me.

☐ Seldom ☐ Sometimes ☐ Fairly Often ☐ Very Often ☐ Always

8. _____ and I agree on what is important for me to work on.

☐ Always ☐ Very Often ☐ Fairly Often ☐ Sometimes ☐ Seldom

9. I feel _____ cares about me even when I do things that he/she does not approve of.

☐ Seldom ☐ Sometimes ☐ Fairly Often ☐ Very Often ☐ Always

10. I feel that the things I do in therapy will help me to accomplish the changes that I want.

☐ Always ☐ Very Often ☐ Fairly Often ☐ Sometimes ☐ Seldom

11. _____ and I have established a good understanding of the kind of changes that would be good for me.

☐ Always ☐ Very Often ☐ Fairly Often ☐ Sometimes ☐ Seldom

12. I believe the way we are working with my problem is correct.

☐ Seldom ☐ Sometimes ☐ Fairly Often ☐ Very Often ☐ Always

Annex D. WAI SR Therapist (English version)

Working Alliance Inventory-Short Revised - Therapist (WAI-SRT)

Instructions: Below is a list of statements about experiences people might have with their client. Some items refer directly to your client with an underlined space -- as you read the sentences, mentally insert the name of your client in place of ___ in the text.

IMPORTANT!!! Please take your time to consider each question carefully.

1. ___ and I agree about the steps to be taken to improve his/her situation.

□	□	□	□	□
Seldom	Sometimes	Fairly Often	Very Often	Always

2. I am genuinely concerned for ___'s welfare.

□	□	□	□	□
Always	Very Often	Fairly Often	Sometimes	Seldom

3. We are working towards mutually agreed-upon goals.

□	□	□	□	□
Seldom	Sometimes	Fairly Often	Very Often	Always

4. ___ and I both feel confident about the usefulness of our current activity in therapy.

□	□	□	□	□
Seldom	Sometimes	Fairly Often	Very Often	Always

5. I appreciate ___ as a person.

□	□	□	□	□
Always	Very Often	Fairly Often	Sometimes	Seldom

6. We have established a good understanding of the kind of changes that would be good for ___.

☐ Always ☐ Very Often ☐ Fairly Often ☐ Sometimes ☐ Seldom

7. ___ and I respect each other.

☐ Seldom ☐ Sometimes ☐ Fairly Often ☐ Very Often ☐ Always

8. ___ and I have a common perception of his/her goals.

☐ Always ☐ Very Often ☐ Fairly Often ☐ Sometimes ☐ Seldom

9. I respect ___ even when he/she does things that I do not approve of.

☐ Seldom ☐ Sometimes ☐ Fairly Often ☐ Very Often ☐ Always

10. We agree on what is important for ___ to work on.

☐ Always ☐ Very Often ☐ Fairly Often ☐ Sometimes ☐ Seldom

Annex E. WAI SR scoring sheet

Scoring Key for the Hatcher-Gillaspy Short Form of the WAI

H&G Item	Original Item#	Scale	Direction
1	25	T	+
2	4	T	+
3	8	B	+
4	30	G	+
5	19	B	+
6	22	G	+
7	23	B	+
8	24	G	+
9	36	B	+
10	16	T	+
11	32	G	+
12	35	T	+

Notes: G=Goal; T=Task; B=Bond
Direction= To derive a scale or total score, simply sum or take the mean of the items

Annex F. WAI SR Paziente (versione Italiana)

Working Alliance Inventory – Breve Modificato (WAI-SR)

Istruzioni: Di seguito è riportato un elenco di istruzioni e domande sulle esperienze che le persone possono avere riguardo la loro terapia o il proprio Medico di Famiglia. Alcune voci, con uno spazio sottolineato, si riferiscono direttamente al tuo Medico di Famiglia. Mentre leggi le frasi, inserisci mentalmente il nome del tuo Medico di Famiglia al posto di ____ nel testo. Pensa alla tua esperienza riguardo la terapia, e decidi quale categoria meglio descrive la tua esperienza.

IMPORTANTE!!! Prendete tutto il tempo necessario per considerare attentamente ogni domanda.

1. Come risultato di queste sessioni mi è più chiaro il modo con cui potrei cambiare.

☐ Raramente ☐ A volte ☐ Abbastanza spesso ☐ Molto spesso ☐ Sempre

2. Quello che sto facendo nella terapia mi dà un nuovo modo di guardare al mio problema.

☐ Sempre ☐ Molto spesso ☐ Abbastanza spesso ☐ A volte ☐ Raramente

3. Credo di piacere a ____.

☐ Raramente ☐ A volte ☐ Abbastanza spesso ☐ Molto spesso ☐ Sempre

4.___ ed io collaboriamo nel fissare gli obiettivi della terapia.

☐ Raramente ☐ A volte ☐ Abbastanza spesso ☐ Molto spesso ☐ Sempre

5.___ ed io ci rispettiamo.

☐ Sempre ☐ Molto spesso ☐ Abbastanza spesso ☐ A volte ☐ Raramente

6.___ ed io lavoriamo di comune accordo sugli obiettivi (**concordati**).

☐ Sempre ☐ Molto spesso ☐ Abbastanza spesso ☐ A volte ☐ Raramente

7. Io credo che___mi apprezza.

☐ Raramente ☐ A volte ☐ Abbastanza spesso ☐ Molto spesso ☐ Sempre

8. _____ ed io siamo d'accordo su quali sono per me le cose importanti su cui lavorare.

☐ Sempre ☐ Molto spesso ☐ Abbastanza spesso ☐ A volte ☐ Raramente

9. Io sento che____si prende cura di me anche quando faccio cose che lui/lei non approva

☐ Raramente ☐ A volte ☐ Abbastanza spesso ☐ Molto spesso ☐ Sempre

10. Ho la sensazione che quello che faccio nella terapia mi aiuta a realizzare i cambiamenti che voglio.

☐ Sempre ☐ Molto spesso ☐ Abbastanza spesso ☐ A volte ☐ Raramente

11._____ ed io abbiamo stabilito una buona comprensione del tipo di cambiamento che sarebbe buono per me.

☐ Sempre ☐ Molto spesso ☐ Abbastanza spesso ☐ A volte ☐ Raramente

12. Io credo che il modo con cui stiamo lavorando sul mio problema sia corretto.

☐ Sempre ☐ Molto spesso ☐ Abbastanza spesso ☐ A volte ☐ Raramente

Annex G. WAI SR Terapista (versione Italiana)

Working Alliance Inventory - Breve Modificato - Terapista (WAI-SRT)

Istruzioni: Di seguito è riportato un elenco di istruzioni riguardo le esperienze che i Medici di Famiglia possono avere con i loro pazienti. Alcune voci, con uno spazio sottolineato, si riferiscono direttamente al paziente. Mentre leggi le frasi, inserisci mentalmente il nome del paziente al posto di ___ nel testo.

IMPORTANTE!!! Prendete tutto il tempo necessario per considerare attentamente ogni domanda.

1.____ed io siamo d'accordo circa i passi da fare per migliorare la sua situazione.

□ □ □ □ □

Raramente A volte Abbastanza spesso Molto spesso Sempre

2. Io sono naturalmente interessato al benessere di_____.

□ □ □ □ □

Sempre Molto spesso Abbastanza spesso A volte Raramente

3. Noi lavoriamo di comune accordo sugli obiettivi concordati.

□ □ □ □ □

Raramente A volte Abbastanza spesso Molto spesso Sempre

4.___ed io siamo sicuri circa l'utilità dell'attività attualmente svolta in ambito terapeutico.

□ □ □ □ □

Raramente A volte Abbastanza spesso Molto spesso Sempre

5. Io apprezzo____come persona

□ □ □ □ □

Sempre Molto spesso Abbastanza spesso A volte Raramente

6. Noi abbiamo stabilito una discreta intesa circa il tipo di cambiamento che andrebbe bene per_____.

□ □ □ □ □

Sempre Molto spesso Abbastanza spesso A volte Raramente

7.____ed io ci rispettiamo.

□ □ □ □ □

Raramente A volte Abbastanza spesso Molto spesso Sempre

8.____ed io abbiamo una comune percezione dei suoi obiettivi.

□ □ □ □ □

Sempre Molto spesso Abbastanza spesso A volte Raramente

9. Io rispetto___anche quando lui/lei fa cose che non approvo.

☐ ☐ ☐ ☐ ☐

Raramente A volte Abbastanza spesso Molto spesso Sempre

10. Noi siamo d'accordo su quali sono per____ le cose importanti su cui lavorare.

☐ ☐ ☐ ☐ ☐

Sempre Molto spesso Abbastanza spesso A volte Raramente

Annex H. Punteggio WAI SR

**Leggenda del punteggio per lo Short Form-Hatcher Gillaspy del WAI SR Patient and Family Physician version**			
	Media scala paziente<br><br>O=Obiettivo=<br>C=Compito=<br>L=Legame=<br><br>Punteggio Totale=	Media scala Medico di Famiglia<br><br>O=Obiettivo=<br>C=Compito=<br>L=Legame=<br><br>Punteggio Totale=	Per ricavare una scala o un punteggio totale, semplicemente fai la somma e dividi per il numero di items.
**WAI SR Item**	**Score Patient version**	**Score Family Physician version**	**Scale type**
1			C
2			C
3			L
4			O
5			L
6			O
7			L
8			O
9			L
10			C
11			O
12			C
